# Patterned Si thin film electrodes for enhancing structural stability

**DOI:** 10.1186/1556-276X-7-20

**Published:** 2012-01-05

**Authors:** Gyu-bong Cho, Jung-pil Noh, Ho-jin Sung, Sang-hun Lee, Yeon-min Im, Hyo-jun Ahn, Ki-won Kim

**Affiliations:** 1 School of Materials Science and Engineering, Research Institute for Green Energy Convergence Technology, Gyeongsang National University, Gazwadong 900, Jinju, Gyeongnam 660-701, Korea

**Keywords:** patterned electrode, silicon film, stress, anode

## Abstract

A patterned film (electrode) with lozenge-shaped Si tiles could be successfully fabricated by masking with an expanded metal foil during film deposition. Its electrochemical properties and structural stability during the charge-discharge process were examined and compared with those of a continuous (conventional) film electrode. The patterned electrode exhibited a remarkably improved cycleability (75% capacity retention after 120 cycles) and an enhanced structural stability compared to the continuous electrode. The good electrochemical performance of the patterned electrode was attributed to the space between Si tiles that acted as a buffer against the volume change of the Si electrode.

## Introduction

The secondary Li-ion batteries with a high energy density have gained attention from wide-range applications of power source for the portable electronics, electric vehicle, and electric storage reservoir. In order to increase the energy density in the limited battery volume, the volume of the cathodic electrode having Li sources should be increased, whereas that of the anodic electrode has to be decreased, that is, anode materials with high theoretical capacity are needed to store the large amount of Li ions.

For the anodic materials, some of the candidates are Si, Sn, Al, Ge, and compounds including these elements [1,2]. Si has a much higher specific energy (4,200 mAh/g for Li_4.4_Si) than commercial graphite (372 mAh/g for LiC_6_). However, there is a severe practical problem in the application of Si electrodes, i.e., when Si is used as an anode material for Li-ion batteries, a large volume expansion/shrinkage occurs during the charge-discharge (lithiation-delithiation) process. The volume change of Si (310%) causes surface cracking and pulverization of the Si film and leads to a rapid capacity fade during initial cycles. The poor electrochemical performances are ultimately caused by repetitive mechanical stress accompanied by large volume changes [3]. Until now, many attempts have been made to prolong the cycle life of Si film electrodes [4-9]. Most researches focused on enhancing the adhesion between the Si film and a current collector (substrate) because the amount of Li storage was limited and the generation of stress was restrained by the enhanced adhesion.

**Figure 1:**
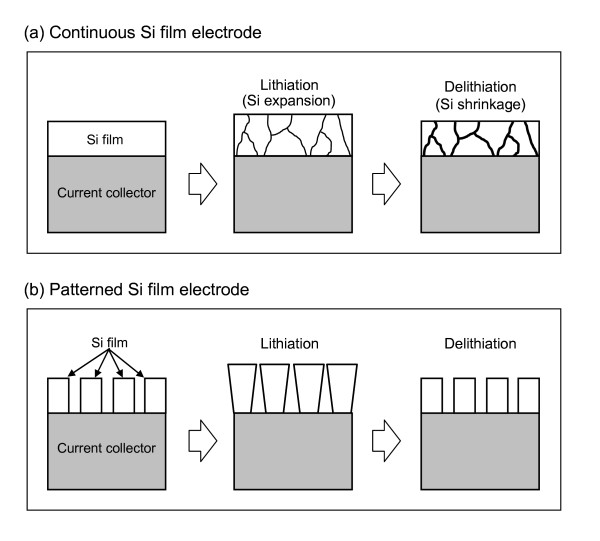
**Schematic diagrams of the structural change in Si film electrodes during the charge-discharge (lithiation-delithiation) process**. (**a**) Continuous film and (**b**) patterned film.

In this work, as a new approach to overcome the problem, space was given to the Si film like a patterned Si film. The Si film including the space is expected to accumulate the stress generated by the volume change during the charge-discharge process. Figure 1 shows schematic diagrams of the estimated changes in Si electrodes with different film types of continuous (conventional) and patterned films during the lithiation-delithiation process. For a continuous Si film, the stress generated by the volume expansion during lithiation generates cracks in the Si film or the interface between the film and substrate, and the volume shrinkage during delithiation causes severe surface cracking [3,10]. In contrast, for a patterned Si film, the space between the patterned Si film units makes room to expand the volume of Si during the lithiation process and to minimize the stress generated in the Si film electrode. These characteristics of the patterned Si film are expected to minimize the structural damage of the Si film and improve the electrochemical reversibility of the electrode.

In this article, the electrochemical properties of the continuous and patterned Si film electrodes are examined, and the improved cycle performance of the patterned electrode is discussed by observing the surface morphologies after 10 cycles.

## Experimental details

A patterned Si film was fabricated by using an expanded metal foil (stainless steel) with lozenge-patterned holes (THANKS-METAL, Japan) as a mask. A continuous (conventional) Si film electrode was also fabricated for comparison. The Si films were deposited on a Cu foil substrate using DC magnetron sputtering systems. Prior to the deposition, the Cu substrate was ultrasonically cleaned and annealed in vacuum-sealed ampules at 573 K for 30 min to remove the residual impurity gases at the surface. The films were grown in a vacuum chamber under a pressure of 5 × 10^-3 ^Torr in argon atmosphere. A cross-sectional analysis was performed to measure thickness of the film with an alpha-step profiler. The thickness of the Si film fabricated in this study was 350 nm.

Crystallinity and surface morphology of the two Si films were investigated by means of transmission electron microscopy [TEM], X-ray diffraction [XRD], and field emission scanning electron microscopy. Although the stress generated during the electrochemical test was indirectly traced by analyzing the broadness of the substrate peaks, a clear distinction before and after the test was difficult.

Electrochemical measurements were preformed in CR2032 coin cells with the different Si film electrodes. A metal lithium foil was used as a counter electrode. Electrolyte was made from 1 M LiPF_6 _in a 1:1 (*v*/*v*) mixture of ethylene carbonate and dimethyl carbonate. The separator used was a porous polypropylene (Celgard 2400; Celgard, Charlotte, NC, USA). Galvanostatic charge-discharge half-cell tests were performed at a current density of 2,100 mA/g (0.5C-rate) at ambient temperature. The test was conducted between the initial OCV and 0.01 V versus Li/Li^+^, then between 0.01 and 1.2 V after the first cycle. Charge-discharge measurements were performed with a constant current. For the calculation of capacity, the mass of the Si electrode is derived from its density, 2.33 g/cm^2^, assuming the crystalline structure.

## Results and discussion

**Figure 2:**
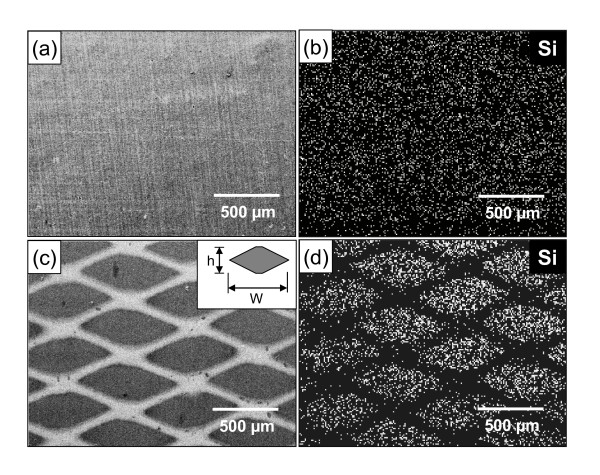
**SEM photographs and EDS mapping images of Si film electrodes**. (**a**, **b**) continuous film and (**c**, **d**) patterned film.

Scanning electron microscopy [SEM] photographs of the continuous and patterned Si films are shown in Figures 2a and 2c, respectively. For the two Si films, energy dispersive spectrometry [EDS] mapping images of the Si element are also given in Figures 2b and 2d, showing the difference in distribution of Si. Tile-like Si films with the lozenge shape observed in the patterned Si film are arranged with the regular space between them. From Figure 2c, the width [w] and height [h] of the Si tile are about 700 μm and 270 μm, respectively, and the Si-deposited area ratio of the patterned film is about 83% for the continuous film. These results demonstrate that a well-patterned Si film (electrode) can be simply fabricated by masking with an expanded metal foil.

**Figure 3:**
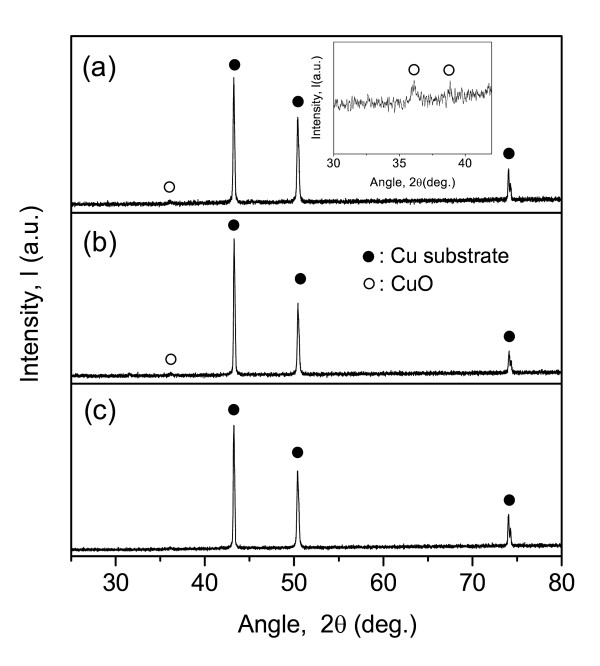
**XRD profiles of (a) Cu current collector (substrate), (b) patterned and (c) continuous Si films**. Partially magnified profile of (a) is in the inset.

Figure 3 shows XRD profiles of continuous and patterned Si films. For comparison, the XRD result of a Cu substrate is presented in Figure 3a. No peaks related to Si can be found for the two films (Figures 3b, c) though the highest-intensity peak of crystalline Si appears at 2*θ *= 28°. This indicates that the Si films fabricated in this work are amorphous. In the previous work, TEM results of the Si film with the same thickness revealed a hollow pattern corresponding to a disordered structure [9]. The amorphous Si is known to be an effective structure to obtain better electrochemical properties than the crystalline Si [7,11].

However, weak peaks of CuO are observed in Figures 3a and 3b. The formation of CuO can be confirmed in the inset of Figure 3a where peaks corresponds to (111) and (200) planes of CuO (JCPDS 80-1719), respectively. The CuO layer seems to form on the surface of the Cu substrate during the annealing process. The intensity of CuO peaks decreases at the patterned Si film and almost disappears at the continuous Si film. This is acceptable because the Si-covered area for the continuous film is wider than that for the patterned film.

**Figure 4:**
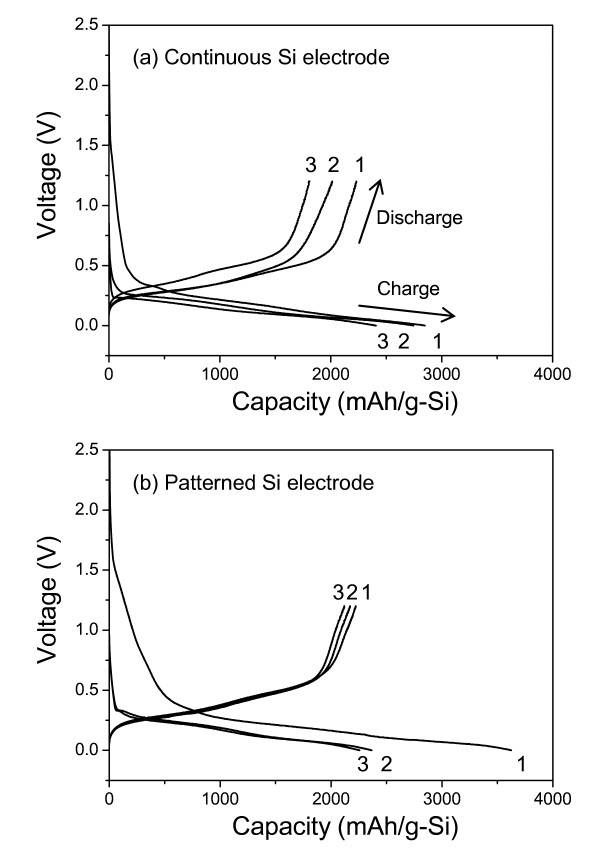
**Initial charge-discharge curves of cells with (a) continuous Si electrode and (b) patterned Si electrode**. Cycle numbers are described at the top and bottom of the curves.

Charge-discharge behaviors of cells with continuous and patterned films (electrodes) are compared in Figure 4. The cell test was performed at the same current density of 2,100 mA/g corresponding to 0.5C-rate. Notice that voltage decreases during the charge process (lithiation). Two voltage plateaus can be observed in each cell, and this is a typical charge-discharge behavior of amorphous Si [12].

At the first cycle, 2,890 mAh/g of charge capacity and 2,200 mAh/g of discharge capacity were obtained from the continuous electrode (Figure 4a), and 3,620 mAh/g and 2,200 mAh/g capacities were obtained from the patterned electrode (Figure 4b). The relatively high charge capacity of the patterned electrode is mainly related to an electrochemical reaction between Li and Cu oxide layers partially exposed on the surface. It had been already reported that the reaction occurred at a voltage range of 1.7 V to 1.0 V and then formed Li_x_CuO [13]. In addition to this, another reason is the solid electrolyte interphase formation that is sensitive to the surface morphology of the electrode because the patterned electrode has a wider surface area than the continuous electrode [6]. These reaction products lead to the capacity loss at the first cycle, and thus a low coulombic efficiency ((discharge capacity/charge capacity) × 100(%)) of 60% was obtained at the first cycle as shown in Figure 4b. However, the patterned electrode exhibits higher efficiencies than those of the continuous electrode which were obtained after the first cycle.

**Figure 5:**
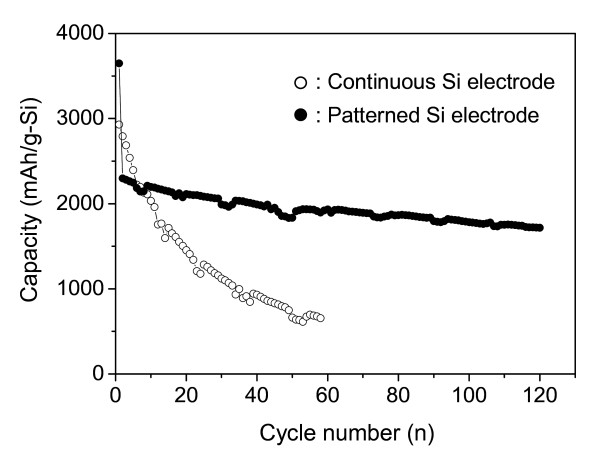
**Charge capacity vs. cycle number curves of cells with continuous and patterned Si electrodes**. The open circle denotes the continuous Si electrode, while the closed circle denotes the patterned Si electrode.

The cycle performances of cells with the continuous and patterned Si electrodes are shown in Figure 5. The charge capacity of the continuous electrode has rapidly decreased within the second cycle. On the other hand, it is noticeable that the patterned Si electrode exhibits a high capacity retention (75% for the second cycle) even until 120 cycles. It is considered that the improved cycle performance of the patterned Si electrode is associated with the stress dispersion in the Si film electrode during the charge-discharge process.

**Figure 6:**
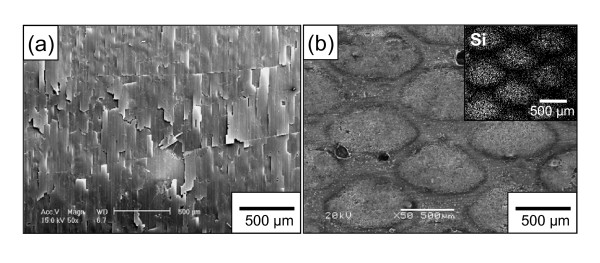
**SEM photographs of cycled Si electrodes**. (**a**) Continuous Si electrode and (**b**) patterned Si electrode. EDS mapping image of the Si element is in the inset of (b).

Figure 6 shows surface morphologies of the continuous and patterned Si electrodes after 10 cycles. The Si film with severe cracks was partially detached from the substrate in the continuous electrode. Such damage of the film electrodes results from the compressive and tensile stress generated by the insertion and extraction of Li [3]. In contrast, Si tiles with the regular space still remained without the severe damage shown in the continuous electrode. It suggests that the stress generated in the continuous Si electrode is larger than that in the patterned electrode.

However, it can be found that the size of the Si tile was slightly increased after cycling (Figure 2b and 6b), and relatively small cracks were generated in the patterned electrode. Unfortunately, these results indicate that the volume change of Si was completely not reversible during the repeated cycling. Therefore, it is concluded that the space between tiles in the patterned Si electrode buffers the volume change of Si during the charge-discharge process and partially disperses the stress generated in the Si electrode. In the next work, it is expected that electrochemical properties of the patterned electrode fabricated on a substrate without an oxide layer will be highly improved because the adhesion between a film and a substrate will be enhanced by the surface treatment of the substrate. Because of this, the study on a surface-etched substrate is in progress.

## Conclusions

A patterned Si film (electrode) with lozenge-shaped tiles could be successfully fabricated by masking with an expanded metal foil, and its electrochemical properties were compared with those of a continuous (conventional) film electrode. The patterned electrode exhibits a remarkably improved cycleability compared to the continuous electrode with 75% capacity retention after 120 cycles. After 10 cycles, the continuous Si film with severe cracks was partially detached from the substrate, whereas Si tiles in the patterned film still remained without severe damage. The good electrochemical performances of the patterned electrode were attributed to the space between Si tiles that acted as a buffer against the volume change of Si.

## Competing interests

The authors declare that they have no competing interests.

## Authors' contributions

GBC, JPN, and YMI participated in the design of the study and carried out the experimental works on SEM, XRD, cycle testing etc. HJS participated in the cycle testing. HJA analyzed the electrochemical data. KWK participated in the drafting of the manuscript. All authors read and approved the final manuscript.
